# Patient Experience and Satisfaction with an e-Health Care Management Application for Inflammatory Bowel Diseases

**DOI:** 10.3390/ijerph182211747

**Published:** 2021-11-09

**Authors:** Aria Zand, Audrey Nguyen, Courtney Reynolds, Ariela Khandadash, Eric Esrailian, Daniel Hommes

**Affiliations:** 1 UCLA Center for Inflammatory Bowel Diseases, Vatche and Tamar Manoukian Division of Digestive Diseases, David Geffen School of Medicine, University of California at Los Angeles, Los Angeles, CA 90095, USA; audrey.d.nguyen@ucla.edu (A.N.); courtney.reynolds@gmail.com (C.R.); akhandad@uci.edu (A.K.); EEsrailian@mednet.ucla.edu (E.E.); d.w.hommes@gmail.com (D.H.); 2 Department of Digestive Diseases, Leiden University Medical Center, 2333 ZA Leiden, The Netherlands

**Keywords:** electronic health, mobile health, mobile applications, chronic disease management, patient experience, inflammatory bowel disease

## Abstract

Background: Rising healthcare expenditures have been partially attributed to suboptimal management of inflammatory bowel diseases (IBD). Electronic health interventions may help improve care management for IBD patients, but there is a need to better understand patient perspectives on these emerging technologies. Aims: The primary aim was to evaluate patient satisfaction and experience with the UCLA eIBD mobile application, an integrative care management platform with disease activity monitoring tools and educational modules. The secondary objective was to capture patient feedback on how to improve the mobile application. Methods: We surveyed IBD patients treated at the UCLA Center for Inflammatory Bowel Diseases. The patient experience survey assessed the patients’ overall satisfaction with the application, perception of health outcomes after participation in the program, and feedback on educational modules as well as areas for application improvement. Results: 50 patients were included. The responses indicated that the patients were greatly satisfied with the ease of patient–provider communication within the application and appointment scheduling features (68%). A majority of respondents (54%) also reported that program participation resulted in improved perception of disease control and quality of life. Lastly, a majority of participants (79%) would recommend this application to others. Conclusions: Mobile tools such as UCLA eIBD have promising implications for integration into patients’ daily lives. This patient satisfaction study suggests the feasibility of using this mobile application by patients and providers. We further showed that UCLA eIBD and its holistic approach led to improved patient experience and satisfaction, which can provide useful recommendations for future electronic health solutions.

## 1. Introduction

Value-based healthcare (VBHC) can be described as the systematic pursuit of the triple aim in healthcare: to improve the individual’s experience, improve health outcomes, and reduce costs [1]. The concept of VBHC is particularly ready for application to long-term management of chronic illnesses since rising healthcare expenditures have been partially attributed to suboptimal management of chronic illnesses, including IBD [2]. The estimated annual disease-attributable cost of IBD is $6.3 billion [3]. Hospitalizations represented over a third of costs, outpatient services—one third. Reducing hospitalization and readmission rates, therefore, continues to be a challenge in chronic disease management. There is clearly an opportunity to reduce costs by increasing the efficiency of outpatient care and preventing hospitalizations.

Electronic health (e-health) interventions are one solution for more effective IBD care management beyond the clinical setting, both in terms of patient outcomes and cost reduction. Smartphone applications are widely available for consumers, and the large population of smartphone users make applications useful tools to manage chronic illnesses like IBD [4]. In fact, smartphone devices with mobile applications and short message reminders have been used effectively by patients with IBD of mild or moderate severity [5].

Furthermore, mobile health technologies have been shown to improve patient outcomes and quality of life [6]. Patient satisfaction with mobile technologies has been observed for many chronic diseases, including asthma [7], HIV [8], diabetes [9], atrial fibrillation [10], and IBD [11]. IBD patients generally have positive views on mobile applications, but there are desirable improvements. A study of Con et al. [11] surveying 86 IBD patients found that 98.8% of the participants were willing to use communication technologies for IBD management, with mobile applications being one of the top two preferred forms. These previous IBD mobile technologies were often created to assess a major single aspect such as quality of life [5], education curriculum [12], or diets [13]. Additional features that patients seek in their chronic disease management applications include easy user interface [14], tracking of disease symptoms [11], and easy access to medical data and services [11].

A systematic assessment of 26 IBD mobile applications found that applications offered a variety of features including diary functionality, pain tracking, bowel movement tracking, and reminders, with application’s content playing a major role in driving patient behavior change [4]. The MyIBD Coach telemedicine tool, which monitors adherence, disease activity, quality of life, and mental health among other measures through validated questionnaires, was shown to be successful, with high rates of patient satisfaction and compliance [15]. It involves collaboration among healthcare providers but is not synchronized with electronic medical records and lacks educational application features on alternative medicine, behavioral health, and physical activity.

To enhance VBHC in IBD, we developed UCLA eIBD to integrate various successful features of previous applications (e.g., appointment reminders, medication trackers) in addition to a healthcare provider portal. UCLA eIBD seeks to provide patients more agency in managing their IBD by increasing their access to healthcare professionals and providing self-help educational modules. Access to care providers through a messaging application provides patients with fast feedback on their conditions and streamlines patient care [16]. The application also contains disease activity, quality of life, and work productivity surveys that facilitate interactions between patients and providers. These tools allow healthcare providers to monitor patients’ disease activity and give direct feedback. This comprehensive application therefore seeks to enhance patient outcomes by including direct connections to the healthcare team and extensive module options.

We previously conducted a pilot study of UCLA eIBD, which found significantly fewer endoscopies and decreases in healthcare utilization, long-term steroid use and IBD-related costs [17]. While it is important to evaluate the efficacy and outcomes of IBD management platforms, it is just as crucial to understand patients’ satisfaction with these platforms to inform their feasibility. Gathering user feedback is necessary to develop the next generation of applications, improve product design, and reduce the gap between application developers and consumers [18,19,20]. This study therefore aims to provide an evaluation of perceived patient satisfaction and experience with the UCLA eIBD mobile application.

## 2. Materials and Methods

### 2.1. Objectives

The primary objective was to measure patient satisfaction and experience with the UCLA eIBD mobile application for care management. The secondary objective was to capture patient feedback on how to improve the mobile application.

### 2.2. Design and Population

We surveyed IBD patients treated at the UCLA Center for Inflammatory Bowel Diseases from October 2017 to October 2018. Included patients were at least 18 years old; diagnosed with Crohn’s disease (CD) or ulcerative colitis (UC) either by means of endoscopy, imaging, or pathology; and had objectively logged into the application in the past year (assessed on the platform). Patients with intestinal cancer, active chemotherapy, or a known intestinal infection were excluded.

All the eligible patients who had logged into the application in the past year were emailed and asked to complete a patient experience survey. Those who did not complete the survey in response to the initial email were followed up and interviewed via phone. No sample size estimation was performed.

### 2.3. Description of UCLA eIBD

**Figure 1:**
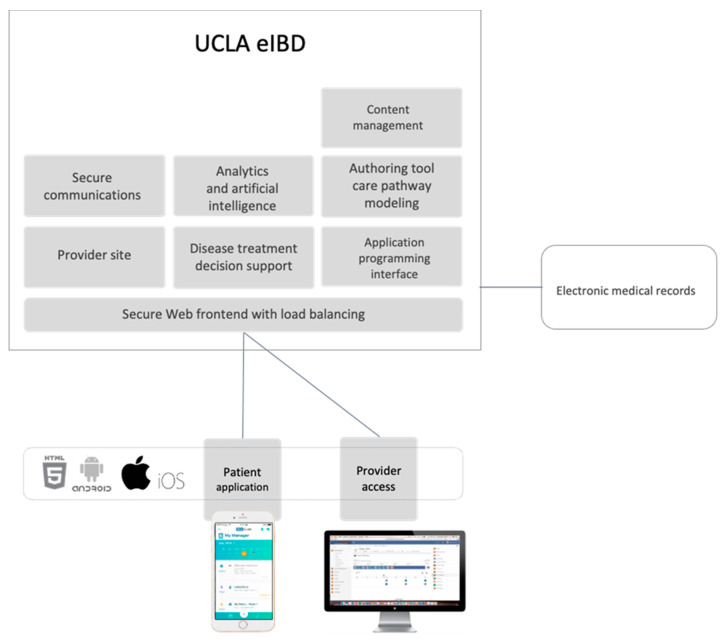
The UCLA eIBD mobile application is an integrative care management platform for patients and providers.

UCLA eIBD is a mobile application that administers a clinic-centered, care management program to its users (Figure 1). It was designed to be a comprehensive tool for patients’ long-term disease management in the IBD outpatient setting. The features of this application include disease activity monitoring, messaging, educational modules, lifestyle modules, and electronic cognitive behavioral therapy (eCBT). The platform is also integrated with UCLA Health’s electronic medical records, allowing patients to view their testing and laboratory results within the application.

For disease activity monitoring, a previously validated tool called the Mobile Health Index was integrated to assess the patients’ disease activity, quality of life, and work productivity [21]. If the surveys indicated poor disease control or a significant change from prior surveys, a message was automatically generated through the application to clinic staff. The enrolled patients could also elect to take these surveys in their own time if they felt they were experiencing a sudden change in their health.

Lastly, the application provided education through several optional interactive modules designed to promote healthy lifestyle habits, including nutrition (My Menu), exercise (My Yoga, My Fitness), relaxation (My Acupressure, My Meditation), and mental health (My Coach). My Menu teaches patients about specific foods to eat and avoid and includes recipes (breakfast, snack, lunch and dinner) designed for IBD patients. My Yoga provides a 6-week program promoting relaxation and flexibility for users. My Acupressure teaches patients about different pressure points for alleviating IBD pain via instructional videos and pictures. My Meditation is a self-guided mindfulness therapy tool that aims to reduce stress-related health issues. My Coach is a personalized mental life coaching program (6-week mental support program) aimed at improving mental well-being and stress management through a cognitive behavioral therapy method.

### 2.4. Data Collection and Outcomes

Patient demographic data were acquired via chart review. The data from the patient experience survey were collected via REDCap [22]. The patient experience survey (Table 1) consisted of 24 items aimed at assessing the patients’ overall satisfaction with the application and their perception of health outcomes after participation in the program. Responses were provided either via a Likert scale or open text. Questionnaire items addressing the application’s features and interface requested feedback on the ease of application use, ability to communicate with staff, and informativeness of modules. Questionnaire items pertaining to the patient’s outcomes asked the patients how effective they felt the application was at improving disease control, work productivity, and quality of life. Lastly, the patients could provide optional open-ended feedback via free text input on ways to improve the application.

## 3. Results

### 3.1. Patient Demographics

In total, 151 patients had been active on the mobile application in the past year, of whom 50 patients responded and completed the survey and thus were included in this study. Regarding the type of IBD, 44% were diagnosed with CD (*n* = 22), 56%—with UC (*n* = 28). Our inclusion cohort had a mean age of 43 years (SD, 14 years) and an average BMI of 25.3 (SD, 6.6). Of the patients, 44% were female, and the majority were White (42%) and non-Hispanic (90%) (Table 2). Most of the patients were non-smokers (78%), and 28% of the patients reported alcohol use. The patients stated use of the following medications: anti-TNF (34%), ASA (16%), combination therapy (32%), IMM (10%), and steroids (6%). Previous abdominal surgeries were reported in 36% of the participants.

### 3.2. Patient Satisfaction

Fifty participants out of the 151 users responded and completed the patient experience survey to provide feedback on the mobile application (Table 1). Responses to the Likert scale questions indicated that the patients were overall satisfied with the patient–provider communication interface of the application. When asked how easy it was to communicate with the program’s staff overall, 52% of the participants responded with “very easy” and 16% responded with “somewhat easy”. A majority of the participants also found it easy to schedule appointments through the application, with 52% and 16% responding with “very easy” and “somewhat easy”, respectively. In addition, a large majority (88%) of the participants reported that the frequency of completing laboratory tests and surveys and scheduling clinic visits was “just right” (Table 1). Regarding the ease of application use, 74% of the participants indicated the application was either “very easy” or “somewhat easy” to navigate.

Additionally, a majority of the participants reported an improved perception of disease control and QoL; 54% of the participants indicated significant or some improvement in their disease control. When asked how program participation affected QoL, 26% indicated significant improvement, 30%—some improvement. Regarding work productivity, 44% indicated significant or some improvement.

When the participants were asked whether they would recommend this application to their friends, family, or other patients on a ten-point scale, with 10 being most likely, the median score was 8, and 79% indicated a score greater than 5. When asked about how informative the application was, 46% of the patients felt that the application was “somewhat” or “very” informative.

### 3.3. Patient Usage of Educational Modules

A majority of the patients completed modules as part of their participation in the program. The most used modules were My Fitness and My Menu (Table 1). Among the patients who participated in the CBT modules (12%), 28% indicated significant or some improvement in their mental health.

When asked about what they liked and disliked about the modules, the patients identified positive aspects to be the modules’ informative content, ease of use, and support of overall well-being (Table 3). For example, one patient said, “They’re easy and I feel great afterwards.” Another patient expressed liking the modules because they “encourage me to take care of my whole self instead of the focus just being on taking my meds”.

The most common reason for not liking the modules was being unsure of the purpose or need for them (8%), particularly for the modules where patients already had their own interventions in place. For example, one patient said they “didn’t feel [the modules] applied to me” while another expressed that they “thought [the module] was good but [I have my] own routine for working out [with regards to My Fitness]”.

### 3.4. Patient Feedback

In the patient experience survey, the patients could provide optional suggestions about additional topics and functionalities they would like the application to cover which were not presently included (Table 4). One participant, for instance, suggested adding a subsection about nutritional advice related to veganism within the My Menu module. Other recommendations included adding a “symptoms tracker”, allowing patients to indicate what symptoms or lack thereof they were experiencing and generating in-application reminders for blood draws or laboratory orders. The other patient-recommended categories to add were the ability to chart laboratory results, side effects of their consequent medications, and gender-specific health topics (Table 4).

The patients’ feedback regarding general comments about the application is also shown in Table 4 (miscellaneous improvement suggestions). One patient stated, “I think this is a great idea and will be very helpful to future patients. I really like being able to communicate with the office without always having to call.” Most patients who provided comments also highlighted aspects that could be improved, such as the application’s interface (e.g., adding a touch ID option to log in; preventing automatic logoff from the application). Other participants reported critical feedback on the application’s content. For instance, one patient stated that the application “is good for people new to IBD, but doesn’t offer as much for people who have had IBD for a while and want more in depth information”.

### 3.5. Summary of Principal Findings

The outcomes suggest that the patients strongly favored the ease of patient–provider communication, with 78% being satisfied. Beneficial outcomes were also seen in patient-reported measures, with 54% reporting a perceived improvement in disease control and 56% reporting a perceived improvement in QoL, indicating that a majority of patients felt the platform positively impacted their health. Additionally, the participants rated this application with a median score out of 10 (10 being most likely) to recommend this application to friends, family, or other patients. My Fitness and My Menu were the two most used optional wellness modules, each reaching the 34% completed status.

## 4. Discussion

### 4.1. Strengths and Comparisons

Our study collected feedback on patient experiences with the UCLA eIBD application after one year of use. Our results could provide guidance for further application development and provide critical feedback for other e-health applications like this one. In fact, mobile tools such as UCLA eIBD have been shown to have promising implications in improving healthcare delivery and integrating into patients’ daily lives. Earlier comparison studies of UCLA eIBD found impacts on costs and healthcare utilization and identified its unique features, such as automated messaging to care coordinators [17,23,24,25]. To complement the previous outcome studies, this study aimed to understand patients’ satisfaction and feedback to help elucidate gaps in the current e-health technologies and inform future designs.

For instance, GI Buddy is a mobile application developed by the Crohn’s Colitis Foundation which enables patients to self-monitor their disease and receive reminders about clinical appointments; however, users cannot directly interact with their providers [26]. Similarly, while the current applications for IBD may be useful for patient monitoring and self-management, many lack professional medical involvement and adherence to clinical guidelines [4]. UCLA eIBD addressed this gap by allowing users to make appointments and message their providers via the platform, in which a majority of users found it “easy” or “very easy” to communicate with their providers. Another self-management tool, myIBD Coach, showed feasibility among patients and providers [15]. As many as 79% of UCLA eIBD users would recommend this application to others (indicated by a score of greater than 5 on the recommendation score item), compared to the 93% found in the myIBD Coach feasibility study [15].

The findings of this novel patient satisfaction study demonstrate the feasibility of UCLA eIBD as a home monitoring tool and some advantages it can provide for both patients and providers. In addition to the patient–provider communication features, the platform’s educational modules are more diverse than the previous tools and provide patients with more alternatives to aid traditional medicine, such as acupuncture, cognitive behavioral therapy, and meditation. These optional modules may improve IBD patients’ well-being and productivity beyond the scope of their disease. For providers, tracking the various modules that the patients use can also provide guidance for tailoring treatment and counseling to the patients’ interests, including nutrition, exercise, and mental health. Lastly, the holistic nature of the application, including features about alternative medicine and assessments for work productivity via the Mobile Health Index, can more completely address the complex, multidimensional factors of chronic disease management.

While the integration of mobile health in IBD management is rapidly expanding, our study also presents novel data from the patient perspective and emphasizes a patient-centered approach towards mobile application development. For instance, the patients’ suggestions to improve the application were centered on specific content interests and the need for additional educational categories (e.g., female health topics), rather than technical problems or lack of need in an application. The fact that the suggestions were less focused on design features could be explained by the overall satisfaction rate of 74% of the participants finding the application easy to navigate. The current and future applications can thus utilize these methods and/or findings to adapt their platforms to address patients’ specific needs, improve satisfaction with their product, and better engage patients in their medical care beyond a doctor’s office visit.

### 4.2. Limitations

Some study limitations should be noted. As the selected patients were individuals who use smartphones, they may be more adept at the usage of applications. Participants were also actively recruited and agreed to participate in this study; thus, a selection bias may have impacted the study results due to the participants being predisposed to wanting to improve their health via e-health solutions. We further acknowledge the sample size was small and relatively homogenous; however, we feel it was adequate for the purpose of directing the future development of this UCLA application and other healthcare applications.

Additionally, the fact that we invited participants to evaluate the application’s feasibility rather than making it mandatory during application usage may explain the response rate of 33%. The response rate should further be considered in the context of challenges associated with adopting e-health technologies into the healthcare space. The obstacles to widespread long-term integration of e-health technologies (e.g., loss of interest, data entry burdens) are still being investigated [27,28]. Despite the growing population of individuals who use mobile health applications, many stop using them over time [29]. Our findings help provide insight to consumer perspectives on application usability and possible explanations to circumvent these challenges.

### 4.3. Future Directions

In an era where the use of mobile technology has become irreplaceable in daily life, there is undoubted benefit of incorporating e-health applications in the management of chronic conditions. Studies have shown proven effect of mobile applications, but also that patients still desire improvements to the existing solutions. We showed that UCLA eIBD and its holistic approach led to greater patient experience and satisfaction, which can provide useful recommendations for healthcare providers and application developers. However, larger and controlled studies are recommended to assess its efficacy at a larger scale and its impact on costs.

## Figures and Tables

**Table 1:** Patient experience survey.

No.	Question	*n* = 50
1	How easy was it to communicate with program staff overall?	26 (52%)—very easy 8 (16%)—somewhat easy 13 (26%)—neutral 3 (6%)—somewhat difficult
2	How easy was it to schedule appointments?	26 (52%)—very easy 8 (16%)—somewhat easy 13 (26%)—neutral 3 (6%)—somewhat difficult
3	How satisfied were you with program staff’s response rate to messages and questions?	22 (44%)—very satisfied 18 (36%)—satisfied 3 (6%)—somewhat dissatisfied 7 (14%)—neutral
4	How did participating in the program affect your disease control?	15 (30%)—significant improvement 12 (24%)—some improvement 21 (42%)—no change 2 (4%)—somewhat worse
5	How participating in the program affect your quality of life?	13 (26%)—significant improvement 15 (30%)—some improvement 20 (40%)—no change 2 (4%)—somewhat worse
6	How did participating in the program affect your work productivity?	11 (22%)—significant improvement 14 (28%)—some improvement 24 (48%)—no change 1 (2%)—somewhat worse
7	Did you participate in the cognitive behavioral therapy modules?	6 (12%)—yes 44 (88%)—no
8	How did participating in the program affect your mental health?	8 (16%)—significant improvement 6 (12%)—some improvement 25 (50%)—no change 1 (2%)—somewhat worse 10 (20%)—unknown
9	Were your clinic visits scheduled too often, just right or not often enough?	44 (88%)—just right 6 (2%)—not often enough
10	Did you feel you were having lab tests done too often, just right or not often enough?	44 (88%)—just right 1 (2%)—not often enough 5 (10%)—too often
11	Did you feel you had to fill out surveys too often, just right or not often enough?	39 (78%)—just right 4 (8%)—not often enough 7 (14%)—too often
12	How accurately do you feel the survey results reflected your opinion of your disease activity and well-being?	17 (34%)—very accurately 20 (40%)—somewhat accurately 11 (22%)—neutral 2 (4%)—somewhat inaccurate
13	How easy was it to navigate the mobile application?	18 (36%)—very easy 19 (38%)—somewhat easy 6 (12%)—neutral 4 (8%)—somewhat difficult 3 (6%)—very difficult
14	Did you find the graphics and overall ‘look’ of the application appealing?	40 (81.63%)—yes 9 (18.37%)—no
15	Overall, how informative was the application, particularly My Academy?	12 (24%)—very informative 11 (22%)—somewhat informative 24 (48%)—neutral 3 (6%)—not informative
16	Which of the following modules did you complete? (choice = My Fitness)	17/50 (34%)
17	Which of the following modules did you complete? (choice = My Meditation)	13/50 (26%)
18	Which of the following modules did you complete? (choice = My Menu)	17/50 (34%)
19	Which of the following modules did you complete? (choice = My Yoga)	10/50 (20%)
20	Which of the following modules did you complete? (choice = My Accupressure)	5/50 (10%)
21	Is there a topic you would like to see added to My Academy or My Wellness? If so, what topic?	Displayed in Table 4.
22	Did you need to access technical support at any time during this study?	7 (14%)—yes 43 (86%)—no
23	If so, how many times did you need to access technical support? *	4 (1 time) 5 (2–5 times)
24	How reliably were you able to reach technical support? *	3 (27%)—somewhat reliable 7 (64%)—neutral 1 (9%)—very unreliable

* Optional question.

**Table 2:** Patient demographics.

Variable	All (*n* = 50)
**Gender**	22 (44%)—female
**Disease Type**	22 (44%)—Crohn’s disease 28 (56)—ulcerative colitis
**Race**	21 (42%)—White 4 (8%)—Black 3 (6%)—Asian 1 (2%)—Armenian 21 (42%)—unknown
**Ethnicity**	4 (8%)—Hispanic 45 (90%)—non-Hispanic 1 (2%)—unknown
**Current smoker**	3 (6%)—current smoker 8 (16%)—former smoker 39 (78%)—never smoker
**Age** (mean SD)	42.58 (SD, 13.6)
**Alcohol use**	14 (28%)—yes 36 (42%)—no
**BMI** (mean SD)	25.3 (SD, 6.6)
**Disease duration** (mean SD)	14.6 (SD, 11.2)
**Disease activity**	29 (58%)—clinical remission 11 (22%)—mild disease activity 6 (12%)—moderate disease activity 3 (6%)—severe disease activity 1 (2%)—unknown
**Medications** - Anti-TNF - ASA - Combination of any medications - IMM - Steroids - No Meds	17 (34%)—anti-TNF 8 (16%)—ASA 16 (32%)—combo 5 (10%)—IMM 3 (6%)—steroids 1 (2%)—no meds
**Abdominal surgeries (%)**	18 (36%)

**Table 3:** The patients’ optional feedback on the modules (*n* = 50). The patients provided open-ended feedback about the educational modules. Their responses were grouped into categories based on the common themes identified across the responses.

**What the Patients Liked about the Modules**	**Count**	**Examples of Patient Feedback**
Informative content	7	“Modules contained useful information.” “My Meditation provided helpful tips.”
Ease of use	3	“Very user friendly.”
Ease of communication with the provider	1	“Liked the VQ visual display. The app gave me comfort because it gave me access to the doctors especially when you have this disease.”
Supports overall well-being	2	“I like that the modules encourage me to take care of my whole self instead of the focus just being on taking my meds.”
Reminders to complete the modules	1	“I like to get reminded to complete the modules, they’re easy and I feel great afterwards.”
The yoga module was simple and effective	1	“I liked the yoga app because it was simple and effective...”
Total	15	
**What the patients disliked about the modules**	**Count**	**Examples of patient feedback**
Not informative	1	“Modules need to contain information that is more specialized.”
Difficult to use	2	“Hard to navigate.”
Unresponsiveness from the staff	1	“Not responsive from staff.”
Did not know about the modules	2	“I did not know about the modules.”
Takes too long to complete	1	“Liked overall content and goal that IBD trying to aim for. Time issue for completing the module.”
Problem with a specific module (My Yoga, My Acupuncture, etc.)	1	“Yoga portion could contain an audio aspect... stopping and reading about doing the yoga was counter-productive to my relaxation.”
Unsure of the purpose or need for them	4	“Didn’t feel like they applied to me, personally.”
Total	12	

**Table 4:** **The patients’ optional feedback on UCLA eIBD**. The patients provided open-text suggestions to improve the application in general. These suggestions were grouped into categories of comment types, including improvements in application content such as possible additional topics and features, as well as miscellaneous critiques.

Comment Types	Total Count	Examples of Patient Feedback (Count)
Suggestions for new application articles and topics	8	A module on acupuncture (1) A module on veganism (1) Side effects of medications (1) Female health topics (1) Blood draw instructions (1) Resources for the recommended pathways (e.g., local places to get nutritional advice, do yoga, fitness) (1) FAQ for family and friends (1)
Suggestions for new application features and tools	3	Ability to chart laboratory results (1) Symptoms tracker (2)
Suggestions for better technical aspects of the application	3	Touch ID for signing in (1) No automatic logoff (1) Different languages (1)
Miscellaneous improvement suggestions	4	Staff response rate faster at the beginning of the program (1) Poor wording of some in-application questionnaires (2) - e.g., *“I don’t like the wording of the questionnaires. i felt they lacked nuance. none asked if i felt overwhelmed, anxious, or preoccupied by disease things. just ‘angry’ or ‘depressed’ which i think are really different experiences.”* - e.g., *“Sometimes i feel just saying on a scale from 1 to 10, how my disease affects my work or social life is too broad a question.”* Lacks in-depth, longer-term information about IBD (1) - e.g., *“app is good for people new to ibd but doesnt offer as much for people who have had ibd for a while and want more in depth information.”*
